# Response rates to a mailed survey of a representative sample of cancer patients randomly drawn from the Pennsylvania Cancer Registry: a randomized trial of incentive and length effects

**DOI:** 10.1186/1471-2288-10-65

**Published:** 2010-07-14

**Authors:** Bridget J Kelly, Taressa K Fraze, Robert C Hornik

**Affiliations:** 1 Research Triangle Institute, Washington, DC, USA; 2 Center of Excellence in Cancer Communication Research, Annenberg School for Communication, University of Pennsylvania, Philadelphia, PA, USA

## Abstract

**Background:**

In recent years, response rates to telephone surveys have declined. Online surveys may miss many older and poorer adults. Mailed surveys may have promise in securing higher response rates.

**Methods:**

In a pilot study, 1200 breast, prostate and colon patients, randomly selected from the Pennsylvania Cancer Registry, were sent surveys in the mail. Incentive amount ($3 vs. $5) and length of the survey (10 pages vs. 16 pages) were randomly assigned.

**Results:**

Overall, there was a high response rate (AAPOR RR4 = 64%). Neither the amount of the incentive, nor the length of the survey affected the response rate significantly. Colon cancer surveys were returned at a significantly lower rate (RR4 = 54%), than breast or prostate surveys (RR4 = 71%, and RR4 = 67%, respectively; p < .001 for both comparisons). There were no significant interactions among cancer type, length of survey and incentive amount in their effects on response likelihood.

**Conclusion:**

Mailed surveys may provide a suitable alternative option for survey-based research with cancer patients.

## Background

In the last several years, researchers have reported declining response rates to telephone surveys across many areas of study [1-5]. While Web-based surveys are becoming increasingly popular as an alternative because they have been shown to result in quicker responses than mailed surveys, average response rates are not high [6]. In addition, their administration can be expensive and the representativeness of many Internet population frames is problematic [7]. There is evidence in the literature that some populations are less likely to use the Internet. For example, those over age 55 are still less likely to use the Internet than their younger counterparts [8,9]. Internet surveys may also miss those of lower socio-economic status [7].

Phone surveys are typically more expensive than either mailed or web surveys, as they require trained interviewers to make the calls. Research comparing modes has found that telephone surveys result in higher likelihood of obtaining extreme positive results due to recency effects [10]. In addition, traditional sampling using RDD methods has faced increasing challenges as more households rely primarily on cell-phones [11,12]. As a result, some research shows that response rates for mailed surveys can be much higher than those administered by phone or Internet [6,13,14].

Given the advantage over web surveys in coverage and the potential cost savings compared to phone surveys, we were interested in using a mailed survey to collect data from patients with three types of cancers. The purpose of the following study was to test the feasibility of using a mailed survey to gather information from recently diagnosed cancer patients, and to test whether specific procedures would affect response rates. We piloted mailed survey methods on a sample of 1,200 cancer patients randomly chosen from the Pennsylvania Cancer Registry and experimented with several strategies to determine how to achieve the highest response rate for the lowest cost. Two different incentive amounts ($3 and $5) and two survey lengths (33 questions--or 10 pages--and 61 questions--or 16 pages) were tested with breast, prostate and colon cancer patients.

## Increasing response rates

Much research has investigated the effects of manipulating specific features (i.e., anonymity; the color; number of follow-up mailings) of a mailed survey to increase response rates or reduce non-response bias [15-28]. One of the most frequently studied features is the inclusion of monetary incentives [17-19]. While there is evidence for the benefit of using incentives [15,17,19-23], researchers have not yet determined an ideal denomination [24]. Some researchers have found that the increase in response rate is not necessarily monotonically related to incentive amount [18,24-26]. Warriner et al. (1996) found $5 to be more effective than $2, but no less effective than $10 [27]. James & Bolstein (1992) showed that response rates increased from $.25 to $.50 and from $.50 to $1, but not between $1 and $2 [18]. In a meta-analysis of randomized controlled trials of monetary incentives, Edwards, Cooper, Roberts and Frost found that the pooled odds ratios for response per $.01 of incentive decreased monotonically as the maximum amount of incentive increased [22]. Dillman provides a theoretical explanation, which suggests that incentive amounts have diminishing returns as the amount approaches the actual value of the service being performed, at which point people perceive answering the survey as more of an economic exchange than a social exchange and have an easier time refusing the money [28].

A second feature manipulated was the length of the survey. Studies on the effect of length on response rates have had mixed results [29]. Some studies, including a review of 200 surveys on patient satisfaction have found no effect [17,30-32]. However, in studies in which the difference between two survey versions was more dramatic, significant effects have resulted [28,33,34]. Two meta analyses of clinical trials found that shorter questionnaires increased the likelihood of response [35,36]. However, Edwards, Roberts, Sandercock & Frost (2004) found that the effects were larger when the questionnaire was short to begin with (which is not the case in this study).

It was also likely that there would be differences in return rates between the three types of cancer. There is not much guidance in the literature about whether patients with one type of cancer might be more likely to respond than others, as the majority of surveys focus on one type of cancer. While a recent study did find that breast cancer patients responded at a higher rate than those with prostate or colon cancer, this study had not been published at the time we were designing our experiment [37]. It is also possible that any of these three factors (incentive, survey length and cancer type) may interact to affect response rates.

And lastly, media coverage of specific cancers, gender differences [38-40], age [39], and disease severity/characteristics [40] are all factors that could potentially affect response rates. Some researchers have argued that the effects of incentives may be contingent upon demographic group [41,42].

Based on the literature and this logic, this study included a number of hypotheses and research questions. The hypotheses included: A $5 incentive will result in higher response rates than a $3 incentive; a longer survey will have a lower response rate than a shorter survey. Research questions included: How will response rates differ by type of cancer? Will length of survey and incentive amount interact with each other or with type of cancer in affecting response rate? Will incentive amount interact with specific demographic characteristics, such as race or marital status?

## Methods

### Participants

**Figure 1:**
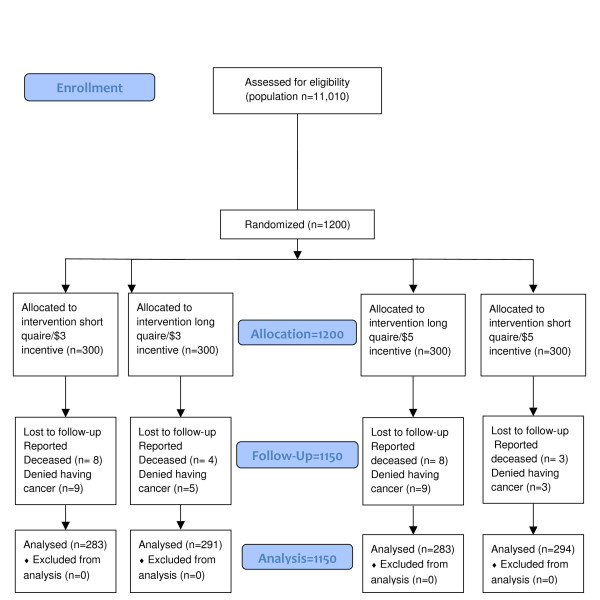
**Consort Flow DiagramTables**.

The sample was drawn from the entire population of patients with cancers of the breast (females only), prostate (males only) or colon (males and females), reported to the Pennsylvania Cancer Registry in 2005 in time to have their data compiled by July of 2006 (approximately 55% of the total number of incident cases in 2005, which was 20,200 across the three cancers; see Figure 1). The age range was not restricted. A sample of 400 people was randomly chosen for each of the three cancers. This was sufficient power to detect differences greater than 5% in response rates between relevant treatment groups (alpha of .05/power at 80%) if the response rate was around 60%.

### Procedure

The design was a two by two, factorial experiment, with incentive amount ($3 vs. $5) and length of the survey (short vs. long) as independent factors. The short version contained 33 questions (10 pages), compared to 61 for the long version (16 pages). The measures excluded from the short version were similar in style and difficulty to those that were retained.

The surveys were designed in Adobe InDesign and printed in one color (blue) with tinting. A glossy cover was added and the result was an $8\frac{1}{2}$ by 11-inch booklet. The content of the surveys for each cancer was identical except for minor wording changes to four of 61 questions. The mailing procedures followed recommendations from Dillman [15]. Procedures were approved by the University of Pennsylvania Institutional Review Board and the Pennsylvania Cancer Registry.

Each cancer patient was randomized to one of four conditions: short survey, $3; short survey, $5; long survey, $3 or long survey, $5. Randomization was conducted using a random number generator in SPSS. Only the research director had access to the master list. Each subject was assigned a code corresponding to one of the four conditions. These codes were placed on the inside back cover of the survey for identification upon their return.

For the incentives, 3 one-dollar bills or 1 five-dollar bill were used. Questionnaire items were packaged by members of the research team and other graduate research assistants who signed confidentiality forms approved by the IRB (as they would be seeing the names and addresses on envelopes).

### Mailing procedure

On September 1^st^, an introductory letter explaining that a survey would be sent in a few days, and a brochure about the Pennsylvania Cancer Registry was mailed. The letter included information indicating that the respondent could opt out of completing the survey, and providing procedures for requesting to be dropped from the study. On September 6th, the first copy of the survey, a second letter, a business reply envelope, and the cash incentive was mailed. The return of the mailed survey served as evidence of consent.

Two weeks later, a reminder letter and another copy of the survey (as well as a business reply envelope) were sent to those from whom no survey had yet been received. No additional cash incentive was included with the third mailing. All mailings were sent via first class mail, to ensure timeliness.

### Measures

#### Dependent variables

*Overall response*. Response rates were calculated four months after the first mailing was sent (in early January), using American Association for Public Opinion Research (AAPOR) standard definitions [43]. For the experimental analyses, AAPOR response rate 2 was used. It allows for the inclusion of both complete and partial questionnaires. Response rate 2 divides the number of questionnaires by the number of refusals and non-refusals. It does not include in the denominator cases that are known to be ineligible -- those who were reported as deceased by a family member (n = 22) or who contacted us to let us know they did not have cancer (n = 27). However, response rate 2 underestimates the true response rate by an unknown amount. It includes in the denominator both all cases which did not return the questionnaire and all cases for whom the post office returned the questionnaires because the addressee was unknown. It is plausible that some of those who did not respond were deceased. To respond to this concern, an adjusted response rate was calculated corresponding to AAPOR's response rate 4, correcting the rate for those estimated to be ineligible to respond, which, in this instance, is reduced by an estimate of those likely to be deceased. Two sources of mortality were taken into account: the expected mortality from all causes given the age distribution of our sample based on CDC published estimates [44], and the incremental mortality specific to their cancer diagnosis and stage. Cancer-specific incremental mortality estimates were based on SEER data on 1- and 2-year survival rates for each of the three cancers [40]. By interpolation, estimates were derived for survival at 16 months, the mean number of months between diagnosis and receipt of the questionnaire (Estimated mortality: 5% for breast, 4% for prostate and 21% for colon). These adjusted response rates (RR4) are reported alongside RR2.

### Statistical Analysis

Results of descriptive analyses presenting response rate by demographic group are shown in Table 1. Summary analyses (presented in Table 2) report RR2 for each condition and RR4 for the three cancers and the total sample. While the overall response rate can be adjusted on the basis of assumptions about mortality among non-respondents, more fine-grained analyses required assigning each case to a response category. Thus, while overall claims about response rates reflect RR4, experimental analyses and demographic group comparisons focus only on these respondents used also for response rate 2. Logistic regression analyses determined the effect of survey length, incentive amount, type of cancer, and interactions among these variables on response rate (Table 3). All regression analyses included three blocks. The first block, the only one reported in Table 3, included the main effects of incentive amount, length of survey, and type of cancer (and for the demographic analyses, all of the demographic variables). Type of cancer was dummy coded with colon cancer patients serving as the comparison group. The second block added the two-way interactions for incentive amount by length of survey, incentive amount by type of cancer, and length of survey by type of cancer (for the demographic analyses, all two-way interactions between these variables and demographics). The third block added the three-way interactions as a forced entry block.

**Table 1:** Demographic characteristics of respondents by condition

	*Short*		*Long*		*χ^2^, pval*	*Total*
						
Demographic	$3	$5	$3	$5		
Age, median (range)	68 (28-93)	68 (29-97)	66 (27-95)	66 (32-94)		67 (27-97)
Female, %	50.5%	53%	48%	50%	1.43, p < .70	50.5%
White race, %	88.8%	90.9%	91.6%	87.8%	25.09, p < .57	89.8%
Hispanic ethnicity, %	1.4%	1.4%	2.3%	1.7%	21.17, p < .27	1.9%
Married, %	62.2%	61.5%	65%	62.4%	13.91, p < .53	62.8%
Stage						
1, %	19.9%	18.8%	21.6%	15.8%	11.97, p < .45	19%
2, %	45%	50%	50%	48%		48%
3, %	13.5%	13%	10%	18%		14%
4, %	9.4	7.4	6.8	7.3		7.7%

^a ^N = 1150

**Table 2:** Response rates by incentive amount, length and cancer type

*Incentive amount*	*Colon* *RR2* *(RR4)*		*Breast* *RR2* *(RR4)*		*Prostate* *RR2* *(RR4)*		*Total* *RR2* *(RR4)*			
	**Short**	**Long**	**Short**	**Long**	**Short**	**Long**	**Short**	**Long**	**Total**	**N**

$3	50.6%	44.9%	69.7%	68.4%	71.6%	63.6%	64.3%	59.4%	61.8%	566
$5	52.1%	41.7%	70.7%	70.7%	69.1%	62.6%	64.1%	58.5%	61.3%	584
Total	47.3%^a^ (57.2%)		69.9%^b^ (73.3%)		66.7%^b^ (69.5%)		64.2%	58.9%	61.6% (67%)	

**Number of usable surveys**	368		392		390		573	577	1150	

^a^Response rates in parentheses are adjusted for estimated deaths, according to CDC and SEER data. Rates with different superscripts were significantly different from each other in logistic regression models.

**Table 3:** Results of logistic regression predicting response from incentive amount, survey length and type of cancer

*Variable*	*Results of logistic regression*					
	**B**	**SE**	**Wald**	**Odds Ratio**	**95% CI**	**p-value**
						
Incentive ($3)	-0.01	0.12	0.01	0.99	(0.78-1.25)	0.92
Survey Length (short)	-0.23	0.12	3.48	0.79	(0.63-1.01)	0.06
Prostate cancer (Colon)	0.81	0.15	28.81	2.24	(1.68-3.02)	< 0.01
Breast cancer (Colon)	0.95	0.15	39.26	2.59	(1.93-3.47)	< 0.01
Constant	0.01	0.14	.01	1.01	(0.77-1.33)	0.92
*Cox and Snell R^2^*	0.043					

^a^n = 1150

^b ^Reference category is in parentheses.

^c^Significant differences in response for type of cancer hold when controlling for age, race, marital status and stage of cancer.

Post hoc analyses were conducted to determine whether those receiving the long survey or the smaller incentive skipped more items than those in the other conditions. Also, for the 33 questions in the short survey, respondents were compared by condition to see if the quality of responses varied. Means and standard deviations were computed for each condition and t-tests conducted to check for significant differences. Additional analyses test differences between responders and non-responders using chi squares and non-parametric tests of medians (for the age variable).

## Results

### Respondent characteristics

Demographics of respondent are presented in Table 1.

### Response rates

The overall estimated RR4 response rate was 67%. The average RR2 rate usable for the experimental analyses across the 12 conditions was 62%, ranging from 42% for colon cancer participants receiving $5 and the long survey to 72% for prostate cancer participants receiving $3 and the short survey (See Table 2).

Logistic regression analyses predicting RR2 response rates revealed no significant differences between participants who received the $3 incentive and participants who received the $5 incentive (OR = .99, p = 0.93) (see Table 3). There was a trend, but not a significant tendency for the short survey length to achieve a higher response rate (OR = .79, p = .06). However, type of cancer did influence response rate. Colon cancer participants responded significantly less often than both breast cancer and prostate cancer participants (*p *< .001 for both comparisons). These differences were still significant when controlling for age, race, marital status and stage of cancer. There were no differences between breast cancer and prostate cancer participants (OR = 1.15, p = .35). None of the two-way interactions - between incentive amount by length; incentive amount by type of cancer; type of cancer by length of survey - nor the three-way interaction for type of cancer by length of survey by amount of incentive were significant (results not shown).

### Demographics and cancer stage

Analyses revealed significant differences for age (p = .002), race (p = .001), and stage of cancer (p = .001) on response rate (see Table 4). Older individuals ( > =65 years) were less likely to respond than younger individuals ( < =64 years; response rates (RR2) were 58% and 67%, respectively). Whites were more likely to respond than non-Whites (RR2 = 64%, compared to 51%). Individuals with metastatic cancer were significantly less likely to respond (RR2 = 44%) than individuals at other stages (RR2 = 66%). There were no significant interactions of incentive amount or length with any of the demographic variables.

**Table 4:** Response rates (%) for demographic groups by condition

*Variable*	*Percentage of total*	*Short/$3*	*Short/$5*	*Long/$3*	*Long/$5*
Race (n = 1,150)					
Non-white	6%	46.4	54.8	45.5	52.9
White	94%	63.8	63.1	57.3	58.8
Gender -colon cancer only (n = 399)					
Female	53%	43.4	47.5	41.3	39.6
Male	47%	46.8	51.3	38.9	40.4
Age (n = 1,138)					
> = 65 Years	54%	57.5	58.4	52.6	51.9
< = 64 Years	46%	66.1	67.8	60.6	65.6
Stage of cancer (N = 1036)					
0	10.9%	65.6	55.6	73.1	64.3
I	19.3%	69.2	67.3	66.1	62.8
II	48.6%	70.4	71.6	65.1	68.2
III	13.8%	64.7	61.8	65.4	46.9
IV	7.3%	42.9	52.6	26.7	52.4

^a^Non-missing n = 1,031. Gender is only presented for colon cancer participants. Results of logistic regression models with interaction terms suggest that none of the variables interacted with condition.

### Post hoc analyses

In order to assess non-response bias, we compared participants who responded and participants who did not on gender (for colon cancer only), age at diagnosis, marital status, race and stage of cancer (see Table 5). Median age for respondents and non-respondents was compared using non-parametric tests of medians. Chi square analyses were conducted for all other variables. The results suggest that despite the high response rate, there were some biases in the sample. This result led us to correct for bias by oversampling stage 4 and African American respondents for subsequent data collection.

**Table 5:** Comparison of respondents and non-respondents on demographic characteristics

	*Colon cancer*		*Prostate cancer*		*Breast cancer*		*Total*	
**Responded?**	**No**	**Yes**	**No**	**Yes**	**No**	**Yes**	**No**	**Yes**

Age (median, SD)	73 (SD = 14)	70.5 (SD = 12)	67 (SD = 10)	68 (SD = 9)	64 (SD = 16)	60 (SD = 13)	70 (SD = 14)	66 (SD = 12)**
Gender (% Female)	51%	53%	NA		NA		NA	
Stage 4	16%	13.5%	7%	2%*	10%	2.3%*	11.5%	5%
Race (% white)	94%	95%	84%	93%*	89%	94%	90%	94%
Marital status (% married)	45%	68%***	70%	85%**	47%	62%*	53%	72%

^a^Non-missing n = 1150.

^b^Significance testing for age was conducted using non-parametric tests of medians. For all other variables, significance testing was conducted using Chi square analyses. *Represent significant differences between respondents and non-respondents on a particular demographic variable. *p < .05; **p < .01; ***p < .001

In addition, analyses were conducted to determine whether survey length or level of incentive was associated with quality of responses, including skipping more questions or answering questions differently. Only 18% skipped any questions. Thus while there was a significant difference by incentive amount in the proportion of people who skipped any questions, the actual number skipped in any condition was so low that there was no practical concern about the difference. There were only chance differences in substantive responses to the questions by incentive and length - examining response to the 33 questions in common across the four surveys.

## Discussion

Overall, the results indicated that a small cash incentive and appropriate recruitment procedures can produce strong response rates to a lengthy mailed survey among cancer patients. Our overall adjusted response rate was 67%. The rates we report here are slightly higher, but comparable to some prior studies, which have achieved response rates of 60-65% [45,46]. However, many of the studies that have achieved the highest response rates have used convenience samples or other non-representative populations. In addition, this study was undertaken more than 10 years after the referenced studies, a period when a decline in response rates to all forms of surveys has been a major concern of researchers. The achieved rate is substantially higher than other studies have found for mailed surveys in the last decade [13,37,47]. In a review of 141 academic papers describing 175 separate studies published in management and behavioral sciences in the years 1975, 1985 and 1995, an average response rate of 55.6% was estimated [47]. Evidence that a mailed survey drawing from a statewide registry in 2006 was able to achieve this rate despite the reported declines in response rates is noteworthy. The only parallel evidence we could find for mailed cancer patient surveys comes from a study in the Netherlands in 2005 using a single hospital's registry, but it also achieved a response rate of 62% [48].

Interestingly, the amount of incentive ($3 versus $5) was not a significant factor predicting response. In fact, the response rates are virtually the same between participants who received $3 and participants who received $5. The finding that the high response rate was received with a relatively small incentive amount is encouraging for future research.

While there was a tendency for the shorter questionnaire to earn a higher response rate, this was not a statistically significant difference. This lack of significant difference was surprising, given the drastic difference between the two surveys. The post hoc results suggest that length and incentive amount did not affect item response either.

The adjusted response rates between breast cancer participants and prostate cancer participants are comparable (73% vs. 69.5%, respectively). However, colon cancer participants responded at a lower rate (57%) than both breast cancer and prostate cancer participants. The pattern among the three cancers is consistent with the large mixed mode ACS survey of survivors, for which 42% of breast, 35% of prostate and 30% of colon cancer patients responded [37]. This pattern of response may be partly explained by the differential level of morbidity associated with these cancers, which may affect ability to respond.

An alternative interpretation relies not on the actual inability to respond but the emotions evoked by the surveys. According to the Leverage-Saliency Theory of Survey Participation, the achieved influence of a particular feature is a function of how important it is to the potential respondent, whether its influence is positive or negative, and how salient it becomes to the sample person during the presentation of the survey request [49]. A survey which reminds a patient that he or she has a cancer with relatively higher morbidity and less positive prognosis (colon cancer) may result in a lower response rate than a survey about a cancer evoking emotions about a health condition with a better prognosis (prostate or breast).

Similarly both of these explanations (actual inability and psychological reluctance) may also explain why patients at more advanced stages of disease were less likely to respond (45% for metastatic versus 66% for others). Colon cancer patients were more likely to be at higher stage at diagnosis (14% metastatic versus 5% for breast and 2% for prostate).

The results by demographics provided some valuable information for the sampling plan in the larger subsequent study. For example, because non-whites and those with stage 4 cancers consistently responded less often across conditions, those two groups were over-sampled in order to ensure sufficient sub-group numbers for the larger study. The lack of evidence for interactions between demographics and condition were encouraging. Even if $3 and short surveys were used, the heterogeneity of the sample would not be compromised.

The high rates of response despite secular declines in response rates may have several explanations. Two possible explanations are discussed here: First, the procedures followed those recommended by Dillman quite closely. Second, these questionnaires were sent to cancer patients who had been diagnosed in the previous calendar year. This was a high salience issue for the patients. Also, the questions that were asked were primarily about their experience in trying to choose treatments and survive with their cancers, and about their use of public information sources, as well as medical sources of information. Respondents may have been appreciative of the opportunity to discuss these topics.

These results are highly generalizable to other cancer patient research contexts given the selection of a representative sample and mailed survey implementation followed standard recommended procedures, but subject to the limitations described below.

## Limitations

The study had several limitations. The fact that the study lacked a $0 incentive control group means it is not possible to conclude that the incentive did not matter, only that there was no difference between the effect of $3 vs. $5 on response rates. The reason for the lack of difference between the $3 and $5 conditions may be that the difference between the two amounts was too small to affect behavior. Had the denominations been $2 and $6 or $0 and $5, there may have been a better chance of detecting differences. A more systematic study would include several different dollar amounts to test differences in the effects between them. However, it is also important to consider cost-effectiveness. For example, a higher dollar amount might result in a slightly higher response rate, but the increase may not justify the additional cost. Future research should investigate the incremental costs per questionnaire returned.

It is possible that the lack of differences reflect cancer patients' sense of altruism or the need to try to give something back to others who might be going through a similar experience. In addition, we know that salience of the survey topic increases the likelihood for response [49]. Leverage-Salience Theory can again explain these findings. In this case, the salience of the survey topic may have been more important than any incentive amount and, thus, diminished the effect of the incentive.

A second limitation is that both response rates exclude twenty-seven people who called or sent letters to say they did not have cancer. It is plausible that some patients who truly do have cancer may deny it, may consider their conditions *pre-cancerous *(such as those with stage 0 colon cancer or ductal carcinoma in situ). It is also possible that some patients may have been mistakenly reported to the PCR (misdiagnosed). In fact, those designated by the PCR as having a Stage 0 cancer did respond at a slightly lower rate than those with stage 1 and stage 2 cancers (58% compared to 65% and 67%, though this was not a significant difference). It is impossible to determine whether any of these people denying the cancer diagnosis may have been misclassified as ineligible. However, this group is 2% of the total sample and thus the effect on the substantive results reported here is negligible.

Despite the high response rates, there is a strong possibility that those least likely to respond to a survey regarding cancer are the people who have fared the poorest in terms of their treatment or who have had the most negative experiences. Groves et al. (2006) write that salience does not always have a positive effect: "When the topic of the survey ... generates negative thoughts, unpleasant memories or reminders of embarrassing personal failings, then the topic may suppress participation despite its personal relevance [3]." When the reasons for non-response are correlated with variables within the survey, non-response bias represents a significant threat to validity. However, the relatively high response rates overall help provide confidence that those biases have been minimized and that a fairly representative sample of the Pennsylvania patient population with these three types of cancer has been included. The lower response rate from stage 4 patients may be an exception to this claim.

## Conclusions

As telephone surveys continue to achieve low response rates and until Web methods can reach a less biased sample, particularly of older and poorer populations, and call on appropriate sampling frames of internet addresses for cancer patients, mailed surveys might provide a promising alternative for reaching cancer patients. The evidence from the survey presented here suggests that even when the burden of questions is high and the incentive amount is small (just $3), high response rates can be achieved.

## Abbreviations

AAPOR American Association for Public Opinion Research. RR2 AAPOR's standard definition for Response Rate 2 allows for the inclusion of both complete and partial questionnaires. Response rate 2 divides the number of questionnaires by the number of refusals and non-refusals. It does not include cases that are known to be ineligible. RR4 AAPOR's Response Rate 4, which reduces the denominator of the RR estimator to eliminate those likely to be ineligible. In this instance the estimate of non-respondents for all reasons is reduced by those likely to be deceased.

## Competing interests

Dr. Kelly works for Research Triangle Institute, a non-profit research institution that provides survey research services.

## Author contributions

BK wrote the background and literature review, conducted some analyses and wrote the discussion section. RH conceived of the idea for the experiment, advised on the approach for response rate calculations and edited and revised several drafts. TF conducted data analyses, provided tables and reviewed drafts. All authors read and approved the final manuscript.

## Pre-publication history

The pre-publication history for this paper can be accessed here:

http://www.biomedcentral.com/1471-2288/10/65/prepub
